# Association of dyslipidemia, diabetes and metabolic syndrome with serum ferritin levels: a middle eastern population-based cross-sectional study

**DOI:** 10.1038/s41598-021-03534-y

**Published:** 2021-12-16

**Authors:** Neyla S. Al Akl, Olfa Khalifa, Khaoula Errafii, Abdelilah Arredouani

**Affiliations:** 1 Diabetes Research Center, Qatar Biomedical Research Institute (QBRI), Hamad Bin Khalifa University (HBKU), Qatar Foundation, Doha, Qatar; 2 College of Health and Life Sciences, Hamad Bin Khalifa University (HBKU), Qatar Foundation, Doha, Qatar

**Keywords:** Diagnostic markers, Predictive markers, Metabolic disorders

## Abstract

Elevated serum ferritin (SFer) levels are implicated in many energy metabolism abnormalities. The association between SFer levels and metabolic disorders has not been studied in Middle Eastern populations. We aimed at exploring the association between SFer levels and serum lipids, diabetes determinants, and metabolic syndrome in a sample of Qatari adults. This study used biochemical parameters obtained from 1928 participants from the Qatar Biobank cohort. We utilized adjusted multivariable logistic regression analysis to estimate the odds ratios (ORs) for dyslipidemia, type 2 diabetes, the homeostasis model assessment of insulin resistance (HOMA-IR), and metabolic syndrome (MetS) according to sex-specific SFer quartiles (Q1 to Q4). Results revealed that the ORs for dyslipidemia increased progressively and significantly across the SFer quartiles, up to two folds in Q4 for women (OR 2.47 (1.68–3.62)) and men (OR 2.24 (1.41–3.55)) versus Q1 (OR:1). Exclusively in women, the ORs for IR (HOMA-IR > 3.58) increased significantly in Q4 (OR 1.79 (1.19–2.70)) versus OR 1 in Q1 as did the ORs for diabetes (OR: 2.03 (1.15–3.57) in Q4 versus OR 1 in Q1). We observed the same result when we pooled the participants with prediabetes and diabetes in one group. The OR for MetS also increased significantly across the Sfer Quartiles from OR: 1 in Q1 to 1.92 (1.06–3.02) in Q4 for women and to 2.07 (1.08–3.98) in Q4 in men. Our results suggest the elevated Sfer levels as a potential risk biomarker for dyslipidemia and MetS in adult Qatari men and women, and diabetes and IR in women only.

## Introduction

In the past three decades, Qatar, and the Gulf Cooperation Council nations in general, have witnessed a quick transition in income status from middle to high. The Gulf state had the highest per capita income in the world in 2018^1^. This rapid economic transition has revolutionized Qataris's lifestyle and caused them to be more prone to develop chronic conditions, such as type 2 diabetes (T2D), dyslipidemia, and metabolic syndrome (MetS)^2^. These conditions are due mainly to the skyrocketing rates of obesity that resulted from the abundance of energy-dense foods and sedentary behavior. Recent statistics from the Qatar Biobank Cohort, which collects extensive lifestyle, clinical, and biological information from adult Qatari nationals and long-term residents from the general population^3^, revealed that about 80% of the adults are overweight or obese^4^. Therefore, it is no surprise to see that about 16% of the adults have T2D^5^, while 33% have dyslipidemia^4^, and 28% satisfy the criteria of MetS^6^.

According to the World Health Organization, the impact of dyslipidemia on mortality, morbidity, and therapeutic expenditures is on the rise^7^, and lipid profile abnormalities are significantly associated with more than 50% of the global burden of ischemic heart disease^8^. On the other hand, the prevalence of T2D and its complications, including nephropathy, neuropathy, retinopathy, hypertension, dyslipidemia, non-alcoholic fatty liver disease, cardiovascular disease, and systemic inflammation, has escalated worldwide^9^. MetS, a condition that encompasses several factors such as central obesity, diabetes, hypertension, and dyslipidemia^10^, also poses a high risk of developing cardiovascular diseases or stroke^6^, and increases the risk of mortality^11^.

Being a key indicator for iron status and serving a pivotal role in iron homeostasis regulation, ferritin is widely studied and is linked to several energy metabolism disorders^12^. Multiple studies have highlighted the association between elevated circulating serum ferritin (Sfer) levels and disturbances in energy metabolism and chronic diseases. In a Chinese study involving 8641 participants, lipid profile dysregulation and dyslipidemia were found to be independently associated with SFer in both men and women, while elevated Sfer was not independently associated with diabetes and insulin resistance (IR)^13^. On the contrary, Forouhi et al.^14^ demonstrated that elevated circulating ferritin level is an independent predictor of diabetes in both men and women; results that correlate with those reported by Akter and colleagues^15^. Also, MetS incidence was higher with elevated SFer levels^16^, and was associated with increasing SFer levels independently in men^17^ and in both sexes^18^.

Despite the findings above, the relationships between serum lipids, diabetes or MetS, and SFer levels remain equivocal. The association between SFer levels and metabolic disorders has not previously been studied in the Middle Eastern populations, where these disorders are highly prevalent. In the present study, we investigated for the first time the association between Sfer levels and metabolism disorders, including dyslipidemia, diabetes and prediabetes, IR (HOMA-IR) and MetS in a sample of Qatari adults from the general population.

## Materials and methods

### Study design and ethical approval

In this cross-sectional study, we utilized clinical and anthropometric data of 1928 participants obtained from the Qatar Biobank (QBB) cohort, a well-phenotyped cohort that recruits adult men and non -pregnant women (aged 18–89 years) from the general population. For the purpose of this study only Qatari nationals were selected^3^. The participants were fasting for more than 6 h at the time of biospecimen collection. The present study was approved by the institutional review board at QBB (IRB number: Ex-2017-RES-ACC-0054-0018). All work was performed in compliance with the ethical standards stated by the declaration of Helsinki. All participants signed informed consent for the use of their data and samples in medical research.

### Lipid parameters and dyslipidemia

Reference values for the lipid parameters were as follows: Triglycerides (TGs) < 1.7 mmol/L^19^, total cholesterol (TC) < 5.2 mmol/L, LDL < 3.4 for both genders; HDL > 1.3 mmol/L for women and > 1mmo/L for men^20^. Participants were categorized as positive for dyslipidemia if they had one or more of the lipid parameters above the reference range.

### Criteria for T2D, prediabetes, and IR

Diabetes was defined based on the ADA criteria. Specifically, participants with diabetes had FPG ≥ 7.0 mmol/L (126 mg/dl), while those with prediabetes had FPG between 5.6 and 6.9 mmol/L^21,22^. Therefore, for the purpose of our analysis, patients were categorized as diabetes, or PreD (Diabetes and Prediabetes). IR was assessed using the Homeostasis Model Assessment of IR (HOMA-IR) equation; HOMA-IR = (fasting insulin (µU/L) × fasting glucose (mmol/L))/22.5^23^. Unfortunately, no population-based studies have been conducted in Qatar or GCC countries to determine the cut-off value of HOMA-IR. Therefore, we calculated HOMA-IR and used the 75th percentile as the cut-off^24^.

### Metabolic syndrome

Evidence of MetS was identified if a participant meets three out of the five risk factors based on the criteria defined by the International Diabetes Federation (IDF). The risk factors are: 1) abdominal obesity (determined by waist circumference (WC) with ethnicity and gender-specific values), 2) hypertriglyceridemia (TGs ≥ 150 mg/dL (1.7 mmol/L) or specific treatment for this lipid abnormality; 3) hyperglycemia (FPG > 5.6 mmol/L) or previously diagnosed T2D; 4 ) raised blood pressure (systolic BP ≥ 130 or diastolic BP ≥ 85 mm Hg) or treatment of previously diagnosed hypertension, and 5) reduced HDL cholesterol (< 40 mg/dL (1.03 mmol/L) in men & < 50 mg/dL (1.29 mmol/L) in women) or specific treatment for this lipid abnormality. However, according to IDF, if BMI is ≥ 30 kg/m^2^, then central obesity is assumed, and no need to include the waist circumference^25^. Therefore, in this study, central obesity was considered when Body mass index (BMI) ≥ 30 kg/m2.

### Statistical analysis

The study participants were stratified by SFer quartiles according to gender. One-way ANOVA was used to compare the means of the different variables across Sfer quartiles for both gender groups, and student t-test was used to compare means of variables between genders. Unadjusted and adjusted multivariable logistic regressions were computed to examine the strength of association between the different traits and the SFer quartiles separately, where Q1 (lowest ferritin quartile) was used as the base reference. The models were adjusted for age, BMI, liver function (based on serum Alanine transaminase (ALT) and Aspartate transaminase (AST levels), and kidney function (based on serum creatinine level). Results are presented as Odds Ratios (OR) with associated 95% confidence intervals (CI). P < 0.05 was considered statistically significant. We used Stata/IC 16.0 ((http://www.stata.com)) to perform all the statistical analyses.

### Ethical approval

The present study was approved by the institutional review boards at Qatar Biobank (IRB number: Ex -2017- RES-ACC -0054-0018). All participants gave written informed consent for their data and biospecimens to be used in medical research.

## Results

### Characteristics of the study population

The baseline characteristics of the 1928 subjects (57% of women) are detailed in Table 1. The mean BMI was significantly higher in women and was in the overweight/obese range. Similar to what has been reported previously in the Qatari population^4^, the prevalence of overweight/obese in our sample was 80.03% and 78.09% in women and men, respectively. The mean FPG and HbA1c% were in the prediabetes range with no significant differences between sexes. The men showed significantly higher ferritin, TGs (mmol/L), and LDL (mmol/L) levels than women, while HDL levels were higher in women (*P* < 0.05). The participants were divided into four subgroups of serum ferritin quartiles based on gender (Table 2) as follow: Q1 (3–30 µg/L), Q2 (31–77 µg/L), Q3 (78–136 µg/L), and Q4 (137–422 µg/L) in men, and Q1 (3–12 µg/L), Q2 (13–27 µg/L), Q3 (28–68 µg/L), and Q4 (69–422 µg/L) for women. The baseline characteristics were compared by gender between the quartiles (Table 2). The average Sfer levels were higher in the men population compared to women in each quartile. The mean levels for TG, TC, and LDL increased while HDL decreased significantly across the Sfer quartiles in both genders. The average FPG and HbA_1c_ levels increased significantly across quartiles in women only (*P* of trends < 0.001). Mean age in women was increasing across the quartiles.

**Table 1:** General and Clinical Characteristics of the participants by gender.

	Women (n = 1102)	Men (n = 826)	*P*-value
Age (years)	40.9 (13.2)	39.9 (12.0)	0.12
Obesity %	46.55%	37.17%	0.000**
Overweight %	33.48%	40.92%	0.001
Diabetes %	12.07%	13.44%	0.633
Prediabetes %	18.97%	21.19%	0.732
BMI (Kg/m2)	30.1 (6.1)	29.1 (5.3)	0.001**
FPG, mmol/L	5.8 (1.9)	5.9 (2.1)	0.12
HBA 1C, %	5.8 (1.1)	5.8 (1.1)	0.56
Insulin, µIU/ml	11.2 (7.7 )	11.4 (7.8)	0.74
HOMA-IR	3.1 (2.9 )	3.2 (3.1 )	0.44
HOMA-B	120.1 (74.7)	117.7 (74.7)	0.48
HDL, mmol/L	1.4 (0.4)	1.3 (0.3 )	< 0.001**
LDL, mmol/L	3.0 (0.8 )	3.1 (0.9)	0.015 *
Triglyceride, mmol/L	1.2 (0.6 )	1.3 (0.7 )	< 0.001 **
T-Cholesterol, mmol/L	5.0 (0.9 )	5.0 (1.0 )	0.86
Albumin, g/l	44.6 (2.7 )	45.3 (2.8 )	< 0.001
Ferritin, µg/l	53.5 (67.5 )	96.8 (86.7 )	< 0.001
SBP (mmHg)	113.9 (15.4 )	116.8 (14.1)	< 0.001
DBP (mmHg)	68.9 (10.3 )	71.2 (10.7)	< 0.001
Pulse	54.8 (17.5 )	55.0 (15.7)	0.84
Waist circumference (cm)	87.3 (14.0)	92.5 (14.0)	< 0.001
Hip circumference (cm)	108.0 (11.4 )	107.0 (10.90)	0.049
Waist Hip Ratio	0.8 (0.1)	0.9 (0.1 )	< 0.001

Values are expressed as mean ( SD) or proportion. *P < 0.05 and **P < 0.01.

**Table 2:** Distribution of baseline characteristics of study subjects according to gender across the serum ferritin quartiles.

	Women					Men					
	Q1 (297)	Q2 (254)	Q3 (279)	Q4 (272)		Q1 (210)		Q2 (203)	Q3 (211)	Q4 (202)	*P* value of Trend
Ferritin ug/l	7.51 ± 2.84	19.62 ± 4.09	44.60 ± 11.67	144.50 ± 80.94		14.78 ± 8.08		52.58 ± 13.32	103.86 ± 17.03	219.28 ± 78.70	< 0.0001
Ferritin range ug/l	3–12	13–27	28–68	69–422		3–30		31–77	78–136	137–422	
Age, years (median)	38	39	42	43.5		40		38	38	39	
Age, years	38 ± 12	39 ± 13	42 ± 12	43 ± 13	**< 0.0001**	40 ± 12		40 ± 12	38 ± 11	40 ± 11.08	0.2221
BMI, Kg/m2	29.68 ± 6.12	30.13 ± 6.40	29.89 ± 6.25	30.67 ± 5.8	0.2610	28.60 ± 5.22		28.5 ± 4.87	29.1 ± 5.79	30.30 ± 5.35	**0.0020**
FPG, mmol/L	5.38 ± 1.33	5.66 ± 1.86	5.89 ± 2.05	6.12 ± 2.31	**0.0001**	5.7 ± 1.89		5.91 ± 2.1	6.0 ± 2.1	5.95 ± 2.17	0.6305
HBA1C, %	5.62 ± 0.78	5.74 ± 1.06	5.82 ± 1.16	5.9 ± 1.27	**0.0280**	5.02 ± 1.25		5.85 ± 1.18	5.84 ± 1.16	5.70 ± 1.19	0.5797
Insulin µIU/ml	10.26 ± 6.23	9.89 ± 6.14	12.50 ± 9.04	12.22 ± 8.63	**0.0001**	10.42 ± 7.4		11.54 ± 9.33	11.38 ± 7.45	12.08 ± 6.93	0.1977
Cpeptide ng/ml	1.93 ± 0.78	1.88 ± 0.75	2.16 ± 0.94	2.21 ± 1.02	**< 0.0001**	1.88 ± 0.87		2.06 ± 0.91	2.17 ± 1.00	2.35 ± 0.90	**< 0.0001**
HOMAR-IR	2.56 ± 2.08	2.66 ± 2.49	3.48 ± 3.5	3.58 ± 3.42	**0.0003**	2.84 ± 2.81		3.34 ± 3.89	3.21 ± 2.91	3.27 ± 2.58	0.2536
HOMA-B	124 ± 64.55	110.41 ± 58.78	123.11 ± 81.34	114.26 ± 72.79	0.4671	114.85 ± 70.25		116.32 ± 77.16	112.69 ± 67.61	123.85 ± 72.69	0.2981
HDL, mmol/L	1.55 ± 0.37	1.51 ± 0.35	1.4 ± 0.32	1.29 ± 0.35	**< 0.0001**	1.46 ± 0.36		1.32 ± 0.34	1.23 ± 0.31	1.17 ± 0.27	**< 0.0001**
LDL, mmol/L	2.81 ± 0.71	3 ± 0.78	3.08 ± 0.88	3.16 ± 0.94	**< 0.0001**	2.88 ± 0.83		3.08 ± 0.94	3.11 ± 0.87	3.36 ± 0.91	**< 0.0001**
TC, mmol/L	4.85 ± 0.81	5.02 ± 0.84	5.03 ± 0.94	5.09 ± 1.02	**0.0106**	4.86 ± 0.92		4.99 ± 1.01	4.95 ± 0.93	5.22 ± 0.99	**0.0020**
TG, mmol/L	1.04 ± 0.51	1.11 ± 0.57	1.24 ± 0.66	1.37 ± 0.71	**< 0.0001**	1.09 ± 0.59		1.30 ± 0.66	1.34 ± 0.7	1.48 ± 0.78	**< 0.0001**
Iron µmol/ml	10.2 ± 5.45	15.01 ± 5.38	15.14 ± 5.2	17.21 ± 5.27	**< 0.0001**	11.90 ± 5.53		16.85 ± 5.43	16.68 ± 5.17	18.65 ± 5.44	**< 0.0001**
WHR	0.78 ± 0.09	0.79 ± 0.08	0.81 ± 0.096	0.83 ± 0.095	**< 0.0001**	0.83 ± 0.09		0.86 ± 0.09	0.86 ± 0.09	0.88 ± 0.08	**< 0.0001**
SBP, mmHg	**112.4 ± 14.8**	113.35 ± 15.7	113.89 ± 15.75	116.53 ± 17.48	**0.0169**	115.58 ± 15.5		118.46 ± 15.34	116.89 ± 13.37	116.63 ± 13.38	0.2403
DBP, mmHg	**69.65 ± 9.69**	68.27 ± 9.84	69.14 ± 11.47	68.58 ± 10.80	0.4264	67.51 ± 10.22		71.56 ± 10.63	72.64 ± 10.04	73.34 ± 11.23	**< 0.0001**
MetS, n	39	56	66	69	**< 0.0001**	47		58	61	57	0.577

Values are presented as range or mean ± SD. BMI: BMI: body mass index; FPG: fasting plasma glucose; HOMA-IR: homeostatic model assessment of IR; HOMA-B: homeostatic model assessment of β-cell function; HDL: high-density lipoproteins; LDL: low-density lipoproteins; TC: total cholesterol; TG: total triglycerides; WHR: wait-to-hip ratio; SBP: Systolic blood pressure; DBP: Diastolic blood pressure; MetS: metabolic syndrome. Significant p values at 5% level are highlighted in bold.

### Association between gender-specific Sfer quartiles and dyslipidemia

**Figure 1:**
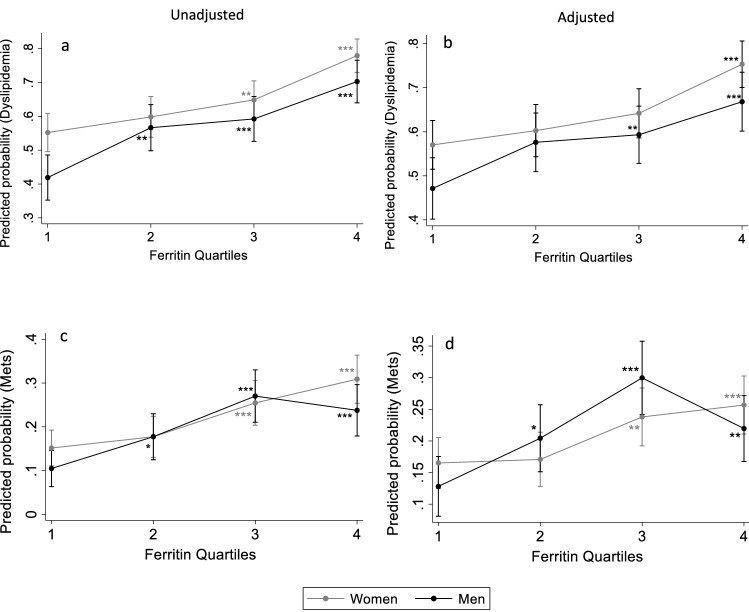
Predicted probabilities for having dyslipidemia or METS across serum ferritin quartiles in men and women. Predicted probabilities for dyslipidemia without adjustments (**a**) and after adjustment for age, BMI and liver and renal factors (**b**). Predicted probabilities for MetS without adjustments (**c**) and after adjustment for age and liver and renal fators (**d**). *, **, ***Indicate statistical significance at respectively 5%, 1% and 0.1%, relative to Q1. The bars represent 95% confidence interval.

To assess the relationship between ferritin levels and dyslipidemia, OR for the occurrence of dyslipidemia was estimated for both men and women across the SFer quartiles (Table 3). Without any adjustment (model 1), the ORs for the risk of dyslipidemia increased progressively and significantly across quartiles in both sexes from 1 in Q1 to 2.86 (1.98–4.13) in Q4 for women, and from 1 in Q1 to 3.28 (2.18–4.93) in Q4 for men, compared to Q1. To determine the independent relationship between Sfer and dyslipidemia and the other metabolic disorders, we adjusted the logistic regression analysis for age, BMI, and liver and renal function. We used categorized values of serum Aspartate Aminotransferase (AST) and Alanine Amino transferase (ALT) for liver function, and serum creatinine for renal function. For normal liver function, ALT was determined between 7 and 56 U/L and AST between 0 and 3U/L^26^. Serum creatine level for normal kidney function was determined between 0.6–1.2 mg/dl for men and 0.5–1.1 mg/dl for women^27^. Age and BMI were used as continuous variables in the adjustment model. The progression of ORs across SFer quartiles remained significant after adjustment (model 2), with OR raising from 1 in Q1 to 2.47 (1.68–3.62) in Q4 in women, and from 1 in Q1 to 2.24 (1.41–3.55) in Q4 in men. We then estimated the dyslipidemia risk probabilities from the unadjusted (Fig. 1a) and adjusted (Fig. 1b) logistic regression models and found that, in both sexes, the probability of having dyslipidemia increases significantly as the Sfer levels increase.

**Table 3:** Odds ratio with 95% CI for the risk of dyslipidemia according to sex-specific serum ferritin quartiles.

	Women				Men			
	Q1 (297)	Q2 (254)	Q3 (279)	Q4 (272)	Q1 (210)	Q2 (203)	Q3 (211)	Q4 (202)
Ferritin, ug/l	7.51 ± 2.84	19.62 ± 4.09	44.60 ± 11.67	144.50 ± 80.94	14.78 ± 8.08	52.58 ± 13.32	103.86 ± 17.03	219.28 ± 78.70
Model 1	1	1.2 (0.86. 1.69)	1.49 (1.07–2.09)*	2.86(1.98–4.13)*	1	1.18 (1.22–2.67)*	2.01 (1.36–2.97)*	3.28 (2.18–4.93)*
Model 2	1	1.15 (0.81–1.62)	1.36 (0.96–1.92)	2.47 (1.68–3.62)*	1	1.5 (0.99–2.28)	1.63 (1.06–2.51)*	2.24 (1.41–3.55)*

Q1: Quartile 1, Q2: Quartile 2, Q3: Quartile 3, Q4: Quartile 4.

Model 1: ORs with no adjustments.

Model 2: OR for Model 1 adjusted for age and BMI, ALT, AST and creatinine.

Significant differences in ORs compared to Q1 are represented with*.

### Rate of Metabolic Syndrome across gender-specific serum ferritin quartiles

Table 4 shows that the unadjusted ORs of MetS (model 1) increased significantly across the Sfer quartiles in both genders; from 1 in Q1 to 2.5 (1.66–3.76) in Q4 in women, and from 1 in Q1 to 2.66 (1.54–4.6) in Q4 in men. This positive relationship remained significant across the Sfer quartiles after adjustment for age and liver and renal functions in model 2; Q4 1.92 (1.22–3.02) in women and Q4 2.07 (1.08–3.98) in men versus OR1 in Q1(Table 4). In men, the highest OR of MetS was observed in Q3 in both models (OR: 3.16 (1.85–5.40) for model1, and OR 3.44 (1.86–6.38 for model2). Like dyslipidemia, the predicted probabilities of having MetS estimated from the unadjusted (Fig. 1c) and adjusted (Fig. 1d) logistic regression models increased significantly across quartiles for men and women.

**Table 4:** Odds ratio of the risk of MetS across sex-specific serum ferritin quartiles.

	Women				Men			
	Q1 (297)	Q2 (254)	Q3 (279)	Q4 (272)	Q1 (210)	Q2 (203)	Q3 (211)	Q4 (202)
Ferritin, ug/l	7.51 ± 2.84	19.62 ± 4.09	44.60 ± 11.67	144.50 ± 80.94	14.78 ± 8.08	52.58 ± 13.32	103.86 ± 17.03	219.28 ± 78.70
Model 1	1	1.20 (0.76–1.89)	1.91(1.26–2.89)*	2.5 (1.66–3.76)*	1	1.84 (1.04–3.25)*	3.16 (1.85–5.40)*	2.66 (1.54–4.6)*
Model 2	1	1.04 (0.64–1.70)	1.67 (1.06–2.63)*	1.92 (1.22–3.02)*	1	1.90 (1.02–3.56)*	3.44 (1.86–6.38)*	2.07 (1.08–3.98)*

Q1: Quartile 1, Q2: Quartile 2, Q3: Quartile 3, Q4: Quartile 4.

Model 1: ORs with no adjustments.

Model 2: OR for Model 1 adjusted for age ALT, AST, and creatinine.

Significant differences in ORs compared to Q1 are represented with*.

### Relationship of Sfer levels with diabetes, PreD, and IR

**Figure 2:**
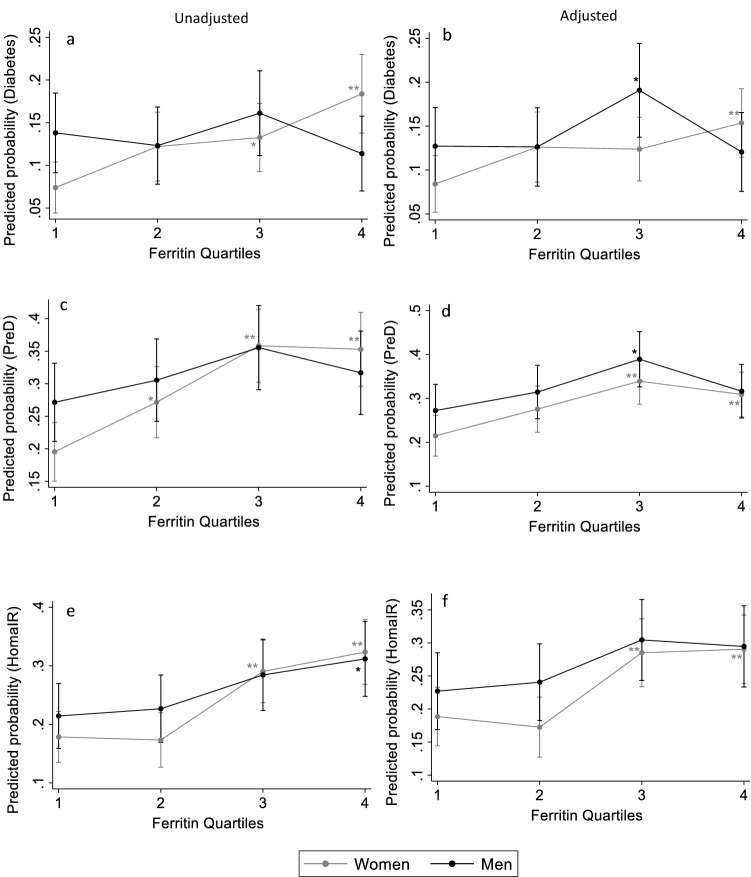
Predicted probabilities for the occurrence of diabetes, PreD or IR across serum ferritin quartiles in men and women. Graphs a, c and e represent the predicted probabilities models without adjustment for Diabetes, PreD and IR respectively. Graphs b, d and f represent the predicted probabilities for the same metabolic conditions after adjustment for age, BMI and liver and renal factors*, **, ***Indicate significant at *P* < 5%, *P* < 1% and *P* < 0.1% respectively relative to Q1. The bars represent 95% confidence interval.

In an attempt to test the association between diabetes, prediabetes, or IR and the increasing SFer levels, unadjusted (model 1 in Table 5) and adjusted (model 2 in Table 5) ORs were estimated. For diabetes (FPG ≥ 7 mmol/l), the OR increases significantly across the Sfer quartiles in women in both models; OR 2.81 (1.65–4.79) in Q4 vs OR:1 in Q1 in model 1 and OR of 2.03 (1.15–3.57) in Q4 vs Q1 OR 1in model 2. Similar significant results were obtained when the participants with prediabetes and diabetes were pooled (FPG ≥ 5.6 mmol/L). . In men, however, the increase in OR across the Sfer quartiles for either diabetes or PreD was not significant. Figure 2illustrates the estimated probabilities from unadjusted (Fig. 2a) and adjusted (Fig. 2b) models of having diabetes in men and women. The unadjusted predicted probabilities increased significantly from 8% ± 1 in Q1 to 15.5% ± 2% in Q4 in women. However, in men, no difference in predicted probability was observed between Q4 (12% ± 2) and Q1 (11% ± 2) . After adjusting the model (Fig. 2b), the increase in predicted probability for diabetes remained significant between Q4 and Q1 in women, but not in men. When prediabetes and diabetes cases were pooled, the estimated probability was higher than diabetes alone across SFer quartiles; probability in Q4 was 31% ± 2 compared to 22% ± 2 in Q1 in women after adjustment (Fig. 2 c and d). Unadjusted and adjusted ORs for the prospects of IR (Table 5) rose significantly in the last quartile in women, OR 1.79 (1.19–2.70) after adjustment compared to OR 1 in Q1. The estimated adjusted probabilities for IR (Fig. 2f), also increaseed significantly between Q1 and Q4 in women ( from 19 ± 2% in Q1 to 29 ± 2% in Q4) in women but not in men.

**Table 5:** Odds ratio with 95% for the risk of diabetes and PreD and IR across sex specific serum ferritin quartiles.

	Women				Men			
	Q1 (297)	Q2 (254)	Q3 (279)	Q4 (272)	Q1 (210)	Q2 (203)	Q3 (211)	Q4 (202)
Ferritin, ug/l	7.51 ± 2.84	19.62 ± 4.09	44.60 ± 11.67	144.50 ± 80.94	14.78 ± 8.08	52.58 ± 13.32	103.86 ± 17.03	219.28 ± 78.70
**Diabetes (FPG ≥ 7 mmol/L)**								
Model1	1	1.73 (0.97:3.08)	1.91 (1.09–3.32)*	2.81 (1.65–4.79)*	1	0.87 (0.49–1.55)	1.19 (0.70–2.05)	0.80 (0.44–1.43)
Model 2	1	1.61 (0.89–2.92)	1.59 (0.89–2.84)	2.03 (1.15–3.57)*	1	1.06 (0.56–2.00)	1.96 (1.05–3.67)*	1.12 (0.56–2.25)
**PreD (FPG ≥ 5.6 mmol/L)**								
Model1	1	1.53 (1.03–2.28)*	2.30 (1.57–3.35)*	2.24 (1.53–3.28)*	1	1.18 (0.77–1.80)	1.48 (0.97–2.24)	1.24 (0.81–1.90)
Model 2	1	1.43 (0.94–2.18)	2.03 (1.36–3.02)*	1.72 (1.15–2.59)*	1	1.32 (0.82–2.13)	2.03 (1.24–3.31)*	1.42 (0.84–2.37)
**HOMAIR > 3.58**								
Model1	1	0.96 (0.62–1.49	1.91 (1.29–2.83)**	2.23 (1.51–3.30)**	1	1.07 (0.67–1.71)	1.49 (0.95–2.32)	1.66 (1.06–2.59)*
Model 2	1	0.90 (0.57–1.41)	1.76 (1.17–2.63)*	1.79 (1.19–2.70)*	1	1.12 (0.68–1.87)	1.59 (0.96–2.65)	1.51 (0.89–2.56)

FPG: fasting plasma glucose; PreD: Prediabetes and Diabetes; HOMA-IR: homeostatic model assessment of IR, Q1: Quartile 1, Q2: Quartile 2, Q3: Quartile 3, Q4: Quartile 4.

Model 1: ORs with no adjustments.

Model 2: OR for Model 1 adjusted for age and B, ALT, AST and creatinine.

Significant differences in ORs compared to Q1 are represented with*.

## Discussion

We investigated the relationship between serum ferritin levels and risk of a set of metabolic disorders, including dyslipidemia, IR, diabetes, prediabetes, and MetS in a sample of adults from Qatar, a Middle Eastern nation, where the prevalence of all these conditions is very high. We found that higher circulating ferritin levels are associated with dyslipidemia and MetS in both men and women. Significant associations were also found with the risk of diabetes, prediabetes, and IR but only in women.

Although iron is an essential molecule that serves as a component in many functional proteins in the body and in oxygen transportation^28^, surplus iron has no direct physiological process for elimination and is stored in ferritin^29^. Elevated SFer levels and their association with disrupted energy metabolism conditions have been studied widely by several groups. Salonen et al.^30^, very early on, addressed the implication of serum ferritin levels on lipid dysregulation and deduced that the risk of myocardial infarction is more than double in men with elevated Sfer levels. Recently, Li et al.^13^ reported the significant association of increasing SFer levels with increasing lipid levels and the risk of dyslipidemia independently of other confounding factors such as age, BMI, diabetes, and lifestyle factors in both men and women. The latter results come in accordance with our findings, detailing the same significant association between increasing Sfer and dyslipidemia regardless of gender. However, since elevated serum ferritin can be due to different aetiologies of which dysfunctional liver or renal function, we adjusted for these factor using levels of liver enzymes ALT and AST and renal product creatinine and found that Sfer is associated with dyslipidemia independent of liver and renal status.

We identified gender differences concerning the association of Sfer levels with hyperglycemia, regardless of whether we use diabetes subjects alone or pool them with those with prediabetes. Our results show that ferritin was associated with hyperglycemia in women only, independently of age, BMI, liver and renal factors. In line with previous work by Nakamura et al.^31^, the association between hyperglycemia and SFer remained significant when we further adjusted for lipid parameters and blood pressure, (data not shown). In contrast Li et al.^13^ found that after adjustment for lipid parameters, the association decreased and became insignificant. These discrepancies warrant further investigation to fully elucidate the relationship between Sfer levels and risk of diabetes.

Previously, it was shown that the association of Sfer levels with risk of IR was independent of other factors in both men and post-menopausal women^32^. Our results partially concurs with these findings. Indeed, we also found that SFer levels were independently associated with increased risk of IR but only in women. It is worth noting that we did not distinguish menopausal and non-menopausal women in our analysis due to the lack of the data. Further investigation is needed to understand this gender-specific association as well as the effect of menopausal state.

MetS has been associated with hyperferritinemia and IR^33^. Leiva et al. reported that the risk of developing MetS was three-fold higher in the highest Sfer quartile group^34^. However, Padwal et al.^12^ documented that the association between MetS and SFer levels was only valid in their male population, while others found that the association was present in post-menopausal women as well^35^. In the present study, we found that the odds of MetS was more than two-fold higher in the highest Sfer quartile in both men and women, as such linking the hyperferritinemia with a higher incidence of MetS in our population. Our results agree with the recent metanalysis published by Zhang et al.^36^, showing that SFer is significantly and positively associated with MetS in both females and males.

The significant associations of Sfer levels with risk of diabetes and abnormalities in lipid parameters suggest that Sfer affects pathways involved in carbohydrate and lipid metabolism and interferes with IR. Although the intertwined pathways linking Sfer to glucose metabolism dysregulation and dyslipidemia is not clearly identified, there are two speculations to determine the underlying pathophysiological mechanisms. The first may be elicited by the release of reactive oxygen species (ROS) by ferritin, leading to lipid peroxidation and directly affecting IR and leading to glucose imbalance^37,38^. The second may be due to lipid accumulation that can be catalyzed by excess ferritin^32^. Indeed, ferritin may block apolipoprotein B secretion^39^ leading to accumulation of cellular triglyceride; high triglyceride levels may deteriorate glucose metabolism due to the release of high concentrations of free fatty acids leading to increased IR^40,41^.

It is worth noting that the association of ferritin with these diseases is not restricted to increased iron level since it has been reported that subjects with MetS and/or IR had hyperferritinemia without having iron overload^33^.

When interpreting our results, some limitations should be considered. Our analyses did not include an adjustment for lifestyle factors such as smoking status, exercising, alcohol consumption, and diet, which could interfere with the regression analysis results and correct the effects of abundant iron, lipid and carbohydrate diet, sedentary lifestyle, and smoking habits. Diabetes was only defined using laboratory cut-off values without any medical history and without distinguishing whether it is type 1 or type 2. History of medication for dyslipidemia or diabetes was not collected, and therefore, no differentiation was measured. However, aside from the above limitations our results are from a well-powered sample of 1928 adult participants from a total of roughly 175,000 eligible adult Qatari nationals. This research is also important because neighboring countries such as Saudi Arabia, the United Arab Emirates, Kuwait, and Bahrain are also impacted by the metabolic illnesses listed above to comparable or even greater degrees than Qatar. Given the cultural, nutritional, behavioral, and ethnical similarities between the peoples of these countries, the current study's findings are likely to apply to them as well.

In conclusion, having unveiled a strong independent association between circulating ferritin and certain metabolic conditions, serum ferritin can be studied as a determinant when evaluating risk of dyslipidemia and MetS in the Qatari population and risk of Diabetes and IR in Qatari women. Furthermore, it would be beneficial to study if this association has a specific orientation concerning premenopausal and post-menopausal status. Further longitudinal cohort studies would convey a more distinct confirmation for our observations.

## Data Availability

The datasets generated during and/or analyzed during the current study are available from the corresponding author on reasonable request.
